# Effect of plasma levels of factor VIII according to procoagulant phospholipids on the risk of future venous thromboembolism

**DOI:** 10.1016/j.rpth.2024.102636

**Published:** 2024-11-26

**Authors:** Magnus S. Edvardsen, Ellen-Sofie Hansen, Thor Ueland, Nadezhda Latysheva, Pål Aukrust, Omri Snir, Vânia M. Morelli, John-Bjarne Hansen

**Affiliations:** 1 Thrombosis Research Group, Department of Clinical Medicine, UiT – The Arctic University of Norway, Tromsø, Norway; 2 Division of Internal Medicine, University Hospital of North Norway, Tromsø, Norway; 3 Research Institute of Internal Medicine, Oslo University Hospital, Rikshospitalet, Oslo, Norway; 4 Institute of Clinical Medicine, Faculty of Medicine, University of Oslo, Oslo, Norway; 5 Section of Clinical Immunology and Infectious Diseases, Oslo University Hospital, Rikshospitalet, Oslo, Norway; 6 Department of Medical Biology, UiT – The Arctic University of Norway, Tromsø, Norway

**Keywords:** factor VIII, phosphatidylserine, phospholipids, venous thromboembolism, venous thrombosis

## Abstract

**Background:**

A high level of plasma coagulation factor (F)VIII is an established and likely causal risk factor for venous thromboembolism (VTE). Procoagulant phospholipids (PPLs) facilitate FVIII activity in coagulation.

**Objectives:**

To assess the association between plasma levels of FVIII and risk of future VTE according to PPL clotting time (PPL_CT_), an inverse surrogate measure of plasma PPL activity.

**Methods:**

A population-based nested case-control study comprising 278 incident VTE cases and 593 randomly selected age- and sex-matched controls were derived from the Tromsø cohort. Exposures were determined from data collected at the cohort baseline. Logistic regression was used to estimate odds ratios (ORs) with 95% CIs for VTE across tertiles of FVIII and PPL_CT_.

**Results:**

High (tertile 3) vs low (tertile 1) FVIII antigen levels resulted in an age- and sex-adjusted OR of 1.53 (95% CI, 0.78-3.00) in those with high PPL_CT_ (low PPL activity), while the corresponding OR for those with low PPL_CT_ (high PPL activity) was 1.88 (95% CI, 0.96-3.66). In the biological interaction analysis, participants with both high FVIII and PPL activity had an OR of 1.86 (95% CI, 0.97-3.57) compared with those with low FVIII and PPL activity. In the joint exposure group, 10% (95% CI, −55% to 75%) of VTEs could be attributable to the interaction between FVIII and PPL activity. Results remained similar after further adjustment for body mass index, C-reactive protein, arterial cardiovascular disease, and cancer.

**Conclusion:**

The effect of high FVIII levels on VTE risk was particularly augmented in those with high PPL activity, suggesting that the effect of FVIII on VTE risk might be partially dependent on PPL activity.

## Essentials

• How plasma FVIII combined with PPL activity affects VTE risk is unknown.
• We assessed the combined effect of high FVIII and PPL activity in a nested case-control study.
• The effect of high FVIII levels on VTE risk was augmented in those with high PPL activity.
• The association between high FVIII and VTE risk might be partially dependent on PPL activity.

## 1 Introduction

Venous thromboembolism (VTE), a disease entity comprising deep vein thrombosis and pulmonary embolism, is a major burden for the affected individuals and society due to severe short- and long-term complications [1]. Further, up to half of VTE events occur in the absence of recognized provoking factors, indicating an inadequate understanding of the pathophysiology [2,3].

A wide body of evidence accumulated over the past decades suggests a critical role of coagulation factor (F)VIII in venous thrombus formation. FVIII circulates in an inactive state bound to von Willebrand factor (VWF) before being cleaved and activated by thrombin when it promotes hemostasis by serving as an important cofactor in the coagulation cascade [4]. Increased plasma levels of FVIII in VTE were first reported in the Leiden Thrombophilia case-control study in 1995 [5]. These findings have later been supported by a significant amount of epidemiologic studies, including prospective studies [6]. Mendelian randomization studies have further implied that the association is causal [7]. However, elevated FVIII levels are typically the result of a concomitant increase in plasma VWF, and the mechanisms linking these proteins with VTE are complex and not fully understood [8]. We have recently reported that high plasma levels of either FVIII or VWF combined with high mean platelet volume (MPV), a measure of increased platelet reactivity, resulted in a particularly high VTE risk and speculated that this occurred due to enhanced coagulation activation at larger platelet surfaces expressing phosphatidylserine [[9], [10], [11]].

The activation of platelets with subsequent exposure of negatively charged procoagulant phospholipids (PPLs), particularly phosphatidylserine, is instrumental in hemostasis as it facilitates the assembly of coagulation factor complexes, such as the tenase complex formed by FIXa and its cofactor, FVIIIa [12]. The availability of PPLs in the circulation is increased considerably upon the release of platelet-derived extracellular vesicles (PDEVs), small double-membrane encapsulated particles derived from the external cellular membrane, which are enriched with negatively charged phospholipids [[13], [14], [15]]. Several studies have reported that elevated plasma extracellular vesicle (EV) levels are associated with increased VTE risk, and it is speculated that this association is largely due to a prothrombotic state caused by PPL exposure on the EV surface [14,16]. Accordingly, we reported a modest association between plasma PPL clotting time (PPL_CT_), an inverse surrogate measure of plasma PPLs, and the risk of incident VTE [17].

Improved insights into FVIII- and EV-dependent mechanisms in venous thrombus formation may facilitate improved strategies for VTE treatment and prevention. To focus on a potential PPL-dependent effect of FVIII on VTE risk, we carried out a nested case-control study derived from the general population where the effect of FVIII levels on the risk of future VTE was assessed across tertiles of PPL_CT_. Our hypothesis was that elevated plasma FVIII would result in a higher VTE risk in the presence of high PPL activity.

## 2 Methods

The fourth survey of the Tromsø Study [18], a Norwegian population-based cohort study, was used to derive a nested case-control study, details of which have been described elsewhere [19]. Briefly, 462 individuals experienced an objectively confirmed incident VTE during follow-up (1994-2007). For each case, 2 age- and sex-matched controls, who were alive at the index date of the VTE, were randomly sampled from the parent cohort (*N* = 924). In total, 515 participants (184 cases and 331 controls) lacked or had plasma samples of insufficient quality (eg, hemolysis). Hence, 278 first-lifetime VTE cases and 593 controls had assessments of both FVIII and PPL_CT_ and were included in the final analyses. All exposures were determined from data obtained at the cohort baseline, and a temporal sequence between exposures and outcome was thus preserved. All participants provided written informed consent, and the study was approved by the regional committee for medical and health research ethics.

Blood samples, physical examinations, and self-administered questionnaires were used to obtain baseline information at Tromsø 4 inclusion (1994-1995). Nonfasting blood samples were collected into EDTA tubes, which were then centrifuged into platelet-poor plasma and stored at −80 °C until analysis. FVIII antigen levels and C-reactive protein (CRP) were measured by enzyme immunoassay, as previously described [9,19]. For FVIII, the intra- and interassay coefficients of variation were <10%. The mean value of FVIII in the control population was defined as 100%, and all values were adjusted accordingly and expressed as percentages [9]. A modified FXa-dependent clotting assay was used to measure PPL_CT_ in EDTA platelet-free plasma, as previously described [17]. The PPL_CT_ displays an inverse and linear association with the level and activity of PPL and EV levels in the samples [20]. The intra- and interassay coefficients of variation were 2.8% and 4.1%, respectively.

STATA version 18.0 (Stata Corporation) was used to perform the statistical analyses. The distributions in the control population were used to determine the tertile cutoffs for plasma FVIII and PPL_CT_. Odds ratios (ORs) for VTE with 95% CIs were calculated by unconditional logistic regression. Within each PPL_CT_ stratum, the reference category comprised those with plasma FVIII in the lowest tertile. Increasing tertiles of FVIII were used to assess *P* for the linear trend of VTE risk. The analyses were adjusted for age and sex in model 1, with the addition of body mass index (BMI) and CRP to model 2, and baseline self-reported history of cancer and arterial cardiovascular disease (CVD) to model 3 to address potential confounding due to mechanisms related to obesity, inflammation, and comorbidities. As in our previous report the association between PPL_CT_ and VTE risk became most notable within extremely prolonged PPL_CT_ (>95th percentile vs ≤25th percentile) [17], we performed sensitivity analysis determining the PPL_CT_ cutoffs at the 80th and 20th percentiles of the control distribution. In the present study, we did not use the 95th percentile as a cutoff due to sample size limitations.

The combined effect of plasma FVIII and PPL_CT_ on VTE risk due to biological interaction was also assessed. For this analysis, the reference category comprised those with concomitant low FVIII and low PPL activity. The biological interaction was assessed by the relative excess risk due to interaction (RERI): OR_AB_ – OR_A_ – OR_B_ + 1 [21], where _A_ and _B_ represent exposure by FVIII and PPL_CT_ in the high-risk category, respectively. The attributable proportion (AP) represents the proportion of VTE events in the combined exposure category, which can be attributed to the interaction between the exposures and corresponds to RERI/OR_AB_ [21]. The RERI and AP were presented with 95% CIs, and values >0 suggest more than additivity, where the effect of the joint exposure on the outcome is greater than the sum of the separate effects.

As the time of follow-up was long in the parent cohort (13 years), results based on a single measurement at the cohort baseline could be influenced by regression dilution bias due to intraindividual fluctuation during the study period [22]. To assess this possibility, we performed regression analyses with a restriction of the maximum time elapsed between baseline blood sampling and the VTE event while keeping all controls in the analyses. Analyses on time restriction were set to require at least 5 VTE events, and ORs adjusted for age, sex, BMI, CRP, and baseline self-reported history of cancer and arterial CVD were generated at every time a new VTE occurred.

## 3 Results and Discussion

Baseline characteristics across tertiles of plasma FVIII levels and PPL_CT_ are summarized in Table 1. Increasing age, BMI, CRP, and proportion of women were observed across increasing tertiles of plasma FVIII, whereas PPL_CT_ was similar across FVIII tertiles. The proportion of self-reported arterial CVD and cancer was higher in tertile 2 and tertile 3 vs tertile 1. There were no substantial differences in the baseline characteristics across PPL_CT_ tertiles.

**Table 1:** Distribution of baseline characteristics according to tertiles of plasma levels of coagulation factor VIII and plasma procoagulant phospholipid clotting time.

Characteristics	FVIII		
	Tertile 1 <85%	Tertile 2 85%-109%	Tertile 3 ≥109%
*n*	261	302	308
Age, y	55 ± 13	62 ± 13	65 ± 13
Sex, male	51.0 (133)	48.0 (145)	43.8 (135)
BMI, kg/m^2^	25.8 ± 3.9	26.3 ± 4.1	27.2 ± 4.7
CRP, mg/L	1.02 (0.51-1.76)	1.15 (0.66-1.87)	1.42 (0.75-2.37)
Cancer^a^	2.3 (6)	6.3 (19)	6.2 (19)
Arterial CVD^a^	12.3 (32)	18.2 (55)	17.5 (54)
PPL_CT_, s	59.7 (50.6-71.5)	58.4 (50.3-71.9)	62.1 (50.8-71.6)

Characteristics	Plasma PPL_CT_		
	Tertile 1 <58.4 s	Tertile 2 58.4-71.1 s	Tertile 3 ≥71.1 s
*n*	297	296	278
Age, y	60 ± 14	61 ± 13	61 ± 14
Sex, male	48.2 (143)	45.3 (134)	48.9 (136)
BMI, kg/m^2^	26.1 ± 4.3	26.8 ± 4.3	26.4 ± 4.2
CRP, mg/L	1.21 (0.65-2.09)	1.07 (0.62-1.91)	1.19 (0.66-2.20)
Cancer^a^	5.1 (15)	6.8 (20)	3.2 (9)
Arterial CVD^a^	14.1 (42)	17.2 (51)	17.3 (48)
FVIII, %	96.7 (81.4-118.9)	99.4 (80.9-118.4)	98.3 (79.8-120.1)

Continuous variables are shown as mean ± SD or median (25th-75th percentile).

Categorical variables are shown as percentages (quantity).

BMI, body mass index; CRP, C-reactive protein; CVD, cardiovascular disease; FVIII, factor VIII; PPL_CT_, procoagulant phospholipid clotting time.

a Self-reported history of cancer, myocardial infarction, angina, or stroke at baseline.

Table 2 shows the ORs for VTE according to tertiles of plasma FVIII in each PPL_CT_ stratum. In participants with high (tertile 3) PPL_CT_ (low PPL activity), high vs low FVIII levels were associated with a 1.5-fold (OR, 1.53; 95% CI, 0.78-3.00) increased age- and sex-adjusted OR for VTE, with a corresponding OR of 1.88 (95% CI, 0.96-3.66) among those with low (tertile 1) PPL_CT_ (high PPL activity). As depicted in Table 3, the combination of low PPL_CT_ (high PPL activity) and high FVIII yielded an age- and sex-adjusted OR for VTE of 1.86 (95% CI, 0.97-3.57) compared with those with low FVIII levels and high PPL_CT_ (low PPL activity). The interaction analyses suggested a RERI of 0.19 (95% CI, −1.03 to 1.41) and an AP of 10% (95% CI, −55% to 75%), implying that 10% of the VTE events in those with high FVIII and low PPL_CT_ (high PPL activity) could be attributed to the interaction, although the 95% CIs of estimates were wide and included zero. Further adjustments for BMI, CRP, and self-reported arterial CVD and cancer (models 2 and 3) had a minor impact on risk estimates and measures of interaction (Tables 2 and 3). In the sensitivity analysis (Supplementary Table), compared with participants with both low FVIII and PPL activity (PPL_CT_ > 80th percentile), those with only high FVIII had an age- and sex-adjusted OR for VTE of 1.14 (95% CI, 0.46-2.80), whereas those with both high FVIII and PPL activity (PPL_CT_ ≤ 20th percentile) had an OR of 1.72 (95% CI, 0.74-4.00). Although not statistically significant, the AP suggested that in the joint exposure group, 33% (95% CI, −40% to 100%) of the VTE events could be attributable to the biological interaction between high FVIII and high PPL activity.

**Table 2:** Odds ratios with 95% CIs for venous thromboembolism across tertiles of factor VIII plasma level within procoagulant phospholipid clotting time stratum.

PPL_CT_, s	FVIII, %	Controls *n* = 593	Cases *n* = 278	Model 1 OR (95% CI)	Model 2 OR (95% CI)	Model 3 OR (95% CI)
T3		196	82			
	T1	65	21	1 (Reference)	1 (Reference)	1 (Reference)
	T2	66	29	1.37 (0.70-2.68)	1.40 (0.71-2.74)	1.40 (0.71-2.76)
	T3	65	32	1.53 (0.78-3.00)	1.46 (0.74-2.88)	1.45 (0.73-2.87)
	*P* for trend			*.2*	*.3*	*.3*
T2		197	99			
	T1	69	19	1 (Reference)	1 (Reference)	1 (Reference)
	T2	59	35	2.24 (1.14-4.39)	2.20 (1.11-4.35)	2.17 (1.09-4.31)
	T3	69	45	2.51 (1.30-4.87)	2.37 (1.21-4.64)	2.37 (1.20-4.67)
	*P* for trend			*.01*	*.02*	*.02*
T1		200	97			
	T1	65	22	1 (Reference)	1 (Reference)	1 (Reference)
	T2	73	40	1.76 (0.93-3.32)	1.77 (0.94-3.35)	1.71 (0.90-3.27)
	T3	62	35	1.88 (0.96-3.66)	1.75 (0.89-3.45)	1.75 (0.87-3.49)
	*P* for trend			*.07*	*.12*	*.13*

Model 1: adjusted for age and sex.

Model 2: adjusted for age, sex, body mass index, and C-reactive protein.

Model 3: adjusted for age, sex, body mass index, C-reactive protein, and self-reported history of cancer and arterial cardiovascular at baseline.

FVIII, factor VIII; OR, odds ratio; PPL_CT_, procoagulant phospholipid clotting time; T, tertile.

**Table 3:** Odds ratios with 95% CIs for venous thromboembolism for the combined effect of factor VIII plasma level and procoagulant phospholipid clotting time.

PPL_CT_, s	FVIII, %	Controls *n* = 593	Cases *n* = 278	Model 1 OR (95% CI)	Model 2 OR (95% CI)	Model 3 OR (95% CI)
T3		196	82			
	T1	65	21	1 (Reference)	1 (Reference)	1 (Reference)
	T2	66	29	1.43 (0.74-2.77)	1.46 (0.75-2.84)	1.45 (0.74-2.83)
	T3	65	32	1.63 (0.84-3.15)	1.52 (0.79-2.96)	1.55 (0.79-3.00)
T2		197	99			
	T1	69	19	0.86 (0.42-1.74)	0.88 (0.43-1.79)	0.88 (0.43-1.79)
	T2	59	35	1.92 (1.00-3.68)	1.91 (0.99-3.67)	1.86 (0.97-3.59)
	T3	69	45	2.16 (1.15-4.04)	2.03 (1.08-3.81)	1.96 (1.04-3.70)
T1		200	97			
	T1	65	22	1.04 (0.52-2.07)	1.07 (0.53-2.13)	1.07 (0.54-2.15)
	T2	73	40	1.76 (0.94-3.30)	1.82 (0.97-3.42)	1.79 (0.95-3.36)
	T3	62	35	1.86 (0.97-3.57)	1.78 (0.93-3.44)	1.75 (0.91-3.38)
RERI (95% CI)				0.19 (−1.03 to 1.41)	0.19 (−1.00 to 1.38)	0.13 (−1.07 to 1.34)
AP (95% CI), %				10 (−55 to 75)	11 (−55 to 77)	8 (−61 to 76)

Model 1: adjusted for age and sex.

Model 2: adjusted for age, sex, body mass index, and C-reactive protein.

Model 3: adjusted for age, sex, body mass index, C-reactive protein, and self-reported history of cancer and arterial cardiovascular at baseline.

AP, attributable proportion; FVIII, factor VIII; OR, odds ratio; PPL_CT_, procoagulant phospholipid clotting time; RERI, relative excess risk due to interaction; T, tertile.

**Figure 1:**
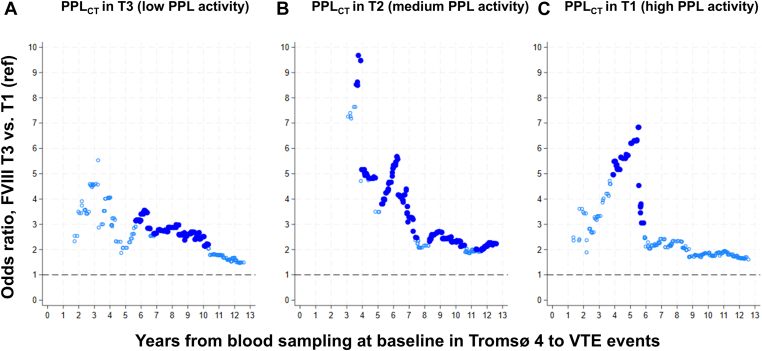
Plots of estimated odds ratios for incident venous thromboembolism (VTE) as a function of time from blood sampling at baseline in Tromsø 4 to VTE events. Participants with plasma factor (F)VIII levels in the highest tertile (T3) were compared with those with FVIII levels in the lowest tertile (T1, reference [ref] category). (A) Analyses of participants with procoagulant phospholipid (PPL) clotting time (PPL_CT_) in T3 (low PPL activity), (B) PPL_CT_ in T2 (medium PPL activity), and (C) PPL_CT_ in T1 (high PPL activity). Analyses were adjusted for age, sex, body mass index, C-reactive protein, and self-reported history of cancer and arterial cardiovascular disease at baseline. Filled circles indicate that risk estimates were statistically significant at a *P* value of <.05.

Next, we estimated ORs considering the time elapsed between baseline blood sampling and the VTE events. The effect on VTE risk of high vs low FVIII levels according to PPL_CT_ tertiles is described in Figure 1. Overall, the ORs pointed toward the null with increasing time between blood sampling and VTE events, denoting a component of regression dilution. The VTE risk for elevated FVIII in those with medium and high PPL activity (Figure 1B, C) was particularly high during the first 5 to 6 years after baseline measurements.

Our results suggest that FVIII, as a strong and robust risk factor for VTE, might be dependent on the presence of high PPL activity in plasma, and this finding seems to be reinforced by the sensitivity analysis when FVIII was particularly associated with an increased OR for VTE only in combination with a more pronounced PPL activity. This is, to our knowledge, the first study to assess the impact of plasma FVIII on VTE risk according to PPL activity. The presence of negatively charged phospholipids in plasma is mainly provided by EVs and is mandatory for the initiation of thrombin generation in plasma *ex vivo* [23,24]. Further, elevated plasma FVIII levels have been shown to shorten the initiation phase and enhance thrombin generation triggered by tissue factor [25]. As parameters of the thrombin generation curve (eg, peak height and endogenous thrombin potential) in plasma *ex vivo* are associated with increased VTE risk [26], it is hypothesized that individuals with high plasma levels of FVIII have a higher potential for thrombin generation in the presence of sufficient PPL.

As FVIII is reliant on negatively charged PPLs to effectively form the intrinsic tenase complex with FIX, attention has been warranted to its interaction with the main expressers of PPLs in the circulation, ie, platelets and PDEVs [12,16]. Interestingly, we previously found a clear synergistic effect of high FVIII and MPV (a marker of platelet size) on VTE risk [9]. As large platelets (high MPV) are more reactive, it may be speculated that a biological interaction between high FVIII and PPLs could partially be provided by large platelets that would be a source of elevated PDEVs with subsequent higher plasma PPL activity. The release of PPL-expressing EVs would also drastically increase the surface available for the assembly of coagulation factors [16]. However, the results on interactions between FVIII and PPLs remained essentially similar after adjustment for MPV (data not shown). Accordingly, even though high MPV is shown to increase platelet adhesivity and aggregability properties, there is no solid proof that larger platelets are more prone to release PDEVs [27,28]. Our data, therefore, suggest that the biological interactions between FVIII and MPV, reflecting FVIII-platelet interaction, and between FVIII and PPLs, reflecting FVIII and EV interaction, on VTE risk are independent of each other.

Some limitations should be addressed. Although the number of baseline plasma samples not available or of inadequate quality for the assessment of FVIII levels and PPL_CT_ was somewhat high, missing data were likely not related to the VTE status, occurring with a similar proportion in VTE cases (40%) and controls (36%). Additionally, baseline characteristics of the study participants with and without measurement of FVIII and PPL_CT_ were similar (data not shown). Thus, the missing data on FVIII and PPL_CT_ were presumably completely at random. Plasma samples were stored for more than 20 years prior to measurements of the biomarkers. However, while this likely leads to differences between true and measured exposure levels, the samples were handled in the same way for cases and controls, and this potential nondifferential misclassification would thereby mainly increase the probability of underestimating the associations. The 95% CIs of estimates derived from the logistic regression and the interaction analyses were wide, reflecting limited statistical power; therefore, caution is needed when interpreting our results.

In conclusion, our findings suggest that the effect of elevated FVIII levels on VTE risk might be higher in those with high PPL activity, particularly during the first few years of follow-up after blood sampling, implying that the impact of FVIII on VTE risk could be partially dependent on PPL activity.
