# Supplementary material for: Effect of plasma levels of factor VIII according to procoagulant phospholipids on the risk of future venous thromboembolism
Source: Res Pract Thromb Haemost. 2024 Nov 26;9(1):102636. doi: 10.1016/j.rpth.2024.102636 (PMC11732527; doi:10.1016/j.rpth.2024.102636)
Supplement: Supplemental material [file mmc1.docx]

**Supplemental material**

**Effect of plasma levels of factor VIII according to procoagulant phospholipids on the risk of future venous thromboembolism**

**Short title:** VTE risk by FVIII and PPL

Magnus S. Edvardsen,^1,2^ Ellen-Sofie Hansen,^1^ Thor Ueland,^1,3,4^ Nadezhda Latysheva,^1^ Pål Aukrust,^3,4,5^ Omri Snir,^1,6^ Vânia M. Morelli,^1,2^ and John-Bjarne Hansen^1,2^

^1^ Thrombosis Research Group (TREC), Department of Clinical Medicine, UiT - The Arctic University of Norway, Tromsø, Norway; ^2^ Division of Internal Medicine, University Hospital of North Norway, Tromsø, Norway; ^3^ Research Institute of Internal Medicine, Oslo University Hospital, Rikshospitalet, Oslo, Norway; ^4^ Institute of Clinical Medicine, Faculty of Medicine, University of Oslo, Oslo, Norway; ^5^ Section of Clinical Immunology and Infectious Diseases, Oslo University Hospital, Rikshospitalet, Oslo, Norway; ^6^ Department of Medical Biology, UiT-The Arctic University of Norway, Tromsø, Norway.

**Supplementary Table 1** Odds ratios with 95% confidence intervals for venous thromboembolism across tertiles of factor VIII plasma level according to 20^th^ and 80^th^ percentiles of procoagulant phospholipid clotting time

|  |  |  |  | Within stratum | | | Combined effects | | |
| --- | --- | --- | --- | --- | --- | --- | --- | --- | --- |
| PPLCT, sec | FVIII, % | Controls | Cases | Model 1  OR (95% CI) | Model 2  OR (95% CI) | Model 3  OR (95% CI) | Model 1  OR (95% CI) | Model 2 OR (95% CI) | Model 3 OR (95% CI) |
| >80th percentile |  | 117 | 42 |  |  |  |  |  |  |
|  | T1 | 34 | 13 | 1 (reference) | 1 (reference) | 1 (reference) | 1 (reference) | 1 (reference) | 1 (reference) |
|  | T2 | 44 | 14 | 0.83 (0.34-2.04) | 0.83 (0.34-2.04) | 0.81 (0.33-2.02) | 0.90 (0.37-2.19) | 0.89 (0.37-2.17) | 0.85 (0.35-2.08) |
|  | T3 | 39 | 15 | 1.00 (0.40-2.52) | 0.92 (0.36-2.36) | 0.92 (0.36-2.36) | 1.14 (0.46-2.80) | 1.02 (0.41-2.56) | 1.00 (0.40-2.50) |
|  | *P* for trend |  |  | 0.98 | 0.88 | 0.87 |  |  |  |
| ≤20th percentile |  | 118 | 60 |  |  |  |  |  |  |
|  | T1 | 44 | 17 | 1 (reference) | 1 (reference) | 1 (reference) | 1.02 (0.43-2.39) | 1.04 (0.44-2.45) | 0.99 (0.42-2.34) |
|  | T2 | 38 | 21 | 1.58 (0.71-3.51) | 1.55 (0.69-3.46) | 1.50 (0.66-3.40) | 1.55 (0.67-3.59) | 1.57 (0.67-3.66) | 1.46 (0.62-3.44) |
|  | T3 | 36 | 22 | 1.76 (0.79-3.93) | 1.72 (0.75-3.92) | 1.72 (0.74-3.99) | 1.72 (0.74-4.00) | 1.69 (0.72-3.96) | 1.61 (0.68-3.81) |
|  | *P* for trend |  |  | 0.17 | 0.20 | 0.21 |  |  |  |
| RERI (95% CI) |  |  |  |  |  |  | 0.57  (-0.74, 1.87) | 0.62  (-0.67, 1.91) | 0.62  (-0.63, 1.87) |
| AP (95% CI) |  |  |  |  |  |  | 33%  (-40%, 100%) | 37%  (-36%, 100%) | 38%  (-36%, 100%) |

PPL_CT_, procoagulant phospholipid clotting time; FVIII, factor VIII; OR, odds ratio; CI, confidence interval; RERI, relative excess risk due to interaction; AP, attributable proportion.

The 20th percentile of PPLCT corresponds to 51.4 sec and the 80th percentile corresponds to 76.4 sec.

Model 1: Adjusted for age and sex.
Model 2: Adjusted for age, sex, body mass index, and C-reactive protein

Model 3: Adjusted for age, sex, body mass index, C-reactive protein, and self-reported history of cancer and arterial cardiovascular at baseline.
